# Virus Safety of Xenotransplantation †

**DOI:** 10.3390/v14091926

**Published:** 2022-08-30

**Authors:** Joachim Denner

**Affiliations:** Institute of Virology, Free University Berlin, 14163 Berlin, Germany; joachim.denner@fu-berlin.de; Tel.: +49-30-838-63059

**Keywords:** xenotransplantation, porcine endogenous retroviruses, porcine viruses, porcine cytomegalovirus, porcine circoviruses, porcine lymphotropic herpesviruses

## Abstract

The practice of xenotransplantation using pig islet cells or organs is under development to alleviate the shortage of human donor islet cells or organs for the treatment of diabetes or organ failure. Multiple genetically modified pigs were generated to prevent rejection. Xenotransplantation may be associated with the transmission of potentially zoonotic porcine viruses. In order to prevent this, we developed highly sensitive PCR-based, immunologicals and other methods for the detection of numerous xenotransplantation-relevant viruses. These methods were used for the screening of donor pigs and xenotransplant recipients. Of special interest are the porcine endogenous retroviruses (PERVs) that are integrated in the genome of all pigs, which are able to infect human cells, and that cannot be eliminated by methods that other viruses can. We showed, using droplet digital PCR, that the number of PERV proviruses is different in different pigs (usually around 60). Furthermore, the copy number is different in different organs of a single pig, indicating that PERVs are active in the living animals. We showed that in the first clinical trials treating diabetic patients with pig islet cells, no porcine viruses were transmitted. However, in preclinical trials transplanting pig hearts orthotopically into baboons, porcine cytomegalovirus (PCMV), a porcine roseolovirus (PCMV/PRV), and porcine circovirus 3 (PCV3), but no PERVs, were transmitted. PCMV/PRV transmission resulted in a significant reduction of the survival time of the xenotransplant. PCMV/PRV was also transmitted in the first pig heart transplantation to a human patient and possibly contributed to the death of the patient. Transmission means that the virus was detected in the recipient, however it remains unclear whether it can infect primate cells, including human cells. We showed previously that PCMV/PRV can be eliminated from donor pigs by early weaning. PERVs were also not transmitted by inoculation of human cell-adapted PERV into small animals, rhesus monkey, baboons and cynomolgus monkeys, even when pharmaceutical immunosuppression was applied. Since PERVs were not transmitted in clinical, preclinical, or infection experiments, it remains unclear whether they should be inactivated in the pig genome by CRISPR/Cas. In summary, by using our sensitive methods, the safety of xenotransplantation can be ensured.

## 1. Introduction

The transmission of a pig virus, the porcine cytomegalovirus/porcine roseolovirus (PCMV/PRV), during the first pig heart xenotransplantation to a human patient [1], demonstrates that aspects of virus safety have been partially neglected in this clinical transplantation. There is no doubt that the virus contributed to the death of the patient, because the pig heart was still functional at the time of death. Clinical features observed in the patient were similar to features that had been described previously in non-human primates receiving pig organs infected with PCMV/PRV. In these preclinical trials, the transmission of PCMV/PRV significantly reduced the survival time of the pig transplant in comparison with transplants free of PCMV/PRV [2,3,4,5,6]. In all cases, it remains unclear whether PCMV/PRV infects primate cells, including human cells. It was proposed that the virus interacts with endothelial and immune cells of the recipient to induce cytokine modulation and defects in coagulation [6]. However, there are good arguments that this accident could be prevented when experienced virologists are involved in future clinical trials [7].

Besides the hepatitis virus E genotype 3 (HEVgt3 or HEV-3), PCMV/PRV is now the second pig virus known to cause a zoonosis in the human recipient. Whether other pig viruses are zoonotic for the human xenotransplant recipient is still unknown. Since comprehensive reviews on PCMV/PRV [2,3,7], HEV [8,9], porcine lymphotropic herpes viruses [10], and porcine endogenous retroviruses (PERVs) [11,12] were recently published in the context of xenotransplantation, this study will concentrate on pig viruses that have not yet been analyzed in such detail.

## 2. Potential Zoonotic Pig Viruses

A zoonosis is an infectious disease caused by a pathogen transmitted from an animal species to humans. Such infectious pathogens may be bacteria, viruses, parasites, or prions. There are many examples of zoonosis in history, the latest is the transmission of the monkeypox virus, which originated from rodents in Africa. The one before was the SARV-CoV-2 pandemic, caused by a corona virus most likely transmitted from bats to humans. The one before that was the transmission of two simian immunodeficiency viruses (SIV), which were apathogenic in their natural hosts. In humans, these viruses, now called humans immunodeficiency viruses 1 and 2 (HIV-1 and HIV-2), induce severe and fatal acquired immunodeficiencies (AIDS).

These recent examples suggest that zoonotic viruses from pigs used as donors for xenotransplantation may be transmitted by the xenotransplant to the (immunosuppressed) recipient. Pigs have been, for many reasons, selected as donor animals. However, it is largely unknown, which of the numerous pig viruses detected in the pig virome can be zoonotic (inducing a disease) for humans. At present, this is known only for HEV-3 and PCMV/PRV.

HEV-3 is widely distributed in wild boars and domestic pigs [13,14,15]. An infection of humans with HEV-3 is, in most cases, the result of consumption of undercooked liver or meat from pork or wild boar, or of direct contact with infected animals [15,16,17]. Acute hepatitis E generally resolves on its own and rarely progresses to acute liver failure or chronic hepatitis, however a majority of HEV infections in immunosuppressed patients, such as organ transplant recipients, progress into chronicity [18]. A vaccine against HEV is available in China, but not in other countries [19], and an HEV-specific antiviral is still lacking, although pegylated interferon and ribavirin have been used to treat chronic HEV infections with mixed results [20].

PCMV/PRV, a roseolovirus closely related to the human herpes viruses 6A, 6B, and 7, is widely distributed in pig populations and most of the infections are sub-clinical [2,3].

The impact of some pig viruses was discussed in numerous reviews (Table 1), however, the potential impact of other viruses was not well analyzed.

The first clinical trials had been performed with islet cells from Auckland Island pigs [44,45,46]. Auckland Island pigs represent an inbred population of feral pigs isolated on the subantarctic island for over 100 years. The animals have been maintained under pathogen-free conditions in New Zealand, they are well characterized virologically (Table 2) [47,48,49], and have been used as donor source in clinical trials, transplanting encapsulated porcine neonatal islet cells for the treatment of human diabetes patients in New Zealand and Argentina. In these trials, no transmission of porcine viruses was observed, including porcine endogenous retroviruses (PERVs) [50,51,52]. Absence of transmission in these cases means that no viral genetic information was found in the recipient.

When a new pig facility was opened at the Center for Innovative Medical Models (CiMM) at the Ludwig Maximilians University, Munich, Germany, the animals were tested for a number of microorganisms (Table 3). All testing is repeated continuously every 6 months on a representative proportion of the current pig population within CiMM to ensure adequate hygiene monitoring [53]. The animals are vaccinated against porcine circovirus 2 (PCV2), porcine parvovirus (PPV), and Erysioelothrix rhusiopathiae. The source animals were made PCMV/PRV-free by early weaning. They were transferred to a commercially available Rescue Deck system dedicated to motherless rearing of piglets and the sows were removed from the facility. The PCMV/PRV status of F1-generation animals was determined by a sensitive real-time PCR-based detection method testing blood, nasal swabs, and cultured peripheral blood mononuclear cells (PBMCs) [53]. This report shows that the elimination of PCMV/PRV from a pig herd is easy to achieve.

In order to generate designated pathogen-free (DPF) pigs to serve as donors for xenotransplantation into clinical patients, a new facility at the Spring Point Project, Minneapolis, MN, USA, was populated with caesarian derived, colostrum deprived piglets. The animals were tested negative for numerous microorganisms (Table 4) [54].

A similar screening was performed with pigs generated for islet cell transplantation at another institution. Screening was performed for more than 30 viruses, including not only PCR-based and immunological methods, but also infection assays and transmission electron microscopy [55]. It is important to note that for some of the pig microorganisms, effective vaccines are available and have been used (Table 5).

The alphaherpesvirus pseudorabies virus (PrV), also called Suid herpesvirus 1 (SuHV-1) or Aujeszky’s disease virus (ADV), is the causative agent of Aujeszky’s disease, an infection of major economic impact in animal husbandry [56]. The disease is highly contagious, transmitted by nose-to-nose contact, and airborne. Eradication of the PrV infection from the national pig populations has been achieved using ‘marker’ vaccines that allow serological differentiation between infected and vaccinated animals. Though the virus is eradicated in domestic swine populations in many countries, it is still present in wild boars [57]. Young pigs are the most severely affected by PrV infection and typically exhibit symptoms of central nervous infection, whereas older swine exhibit symptoms of respiratory disease [56]. PrV can infect a wide variety of mammals, including pigs, sheeps, cattles, etc., thereby causing severe clinical symptoms and acute death [58]. The fact that this virus also infects humans [59] makes it a risk factor in xenotransplantation. This virus is also an excellent example of herpes viruses being not species-specific, and of their ability to cross species barriers.

Other viruses with potential zoonotic potential are the porcine enteric viruses encephalomyocarditis virus (EMCV), the porcine astrovirus (PAstV), the porcine norovirus (PNoV), and the porcine sapovirus (PSaV) [60]. ECMV is another example of pig viruses can overcoming species barrier, for example in zoo outbreaks (for review see [60])**.** Although ECMV infections have been observed in humans, these originated mainly from mice and primates, not from pigs. Alternately, antibodies against ECMV have been found in humans with close contact to pigs, e.g., swine veterinarians. Therefore, pigs may serve as potential reservoirs of transmission of ECMV to humans.

Attention should also be paid to rotaviruses (RVs). Among ten groups of RVs, RV group A (RVA), RV group B (RVB), and RV group C (RVC) show the highest prevalence. Similar to human RVAs, porcine RVAs are widely distributed worldwide. Zoonotic transmission of RVAs have been proven by epidemiological and experimental studies [60]. There are effective vaccines against human RVs and polyvalent vaccines for pigs, e.g., the Prosystem RCE vaccine (Table 5).

Porcine endogenous retroviruses represent a special risk because these viruses are integrated in the genome of all pigs [37]. There are multiple copies in the genome, ranging to 60 or more [39]. It is important to note that the overall number of integrated proviral copies has no significance for the risk posed by these viruses. Only the number of human-tropic infectious viruses is relevant [61]. Most interestingly, the copy number is different in different organs of a single pig, indicating that PERVs are active in living animals [39].

## 3. Detection of Porcine Viruses

There are numerous publications that describe PCR-based, immunological, and other methods to screen for virus infection, especially for some selected xenotransplantation-relevant viruses [62] (Table 6).

The detection systems used for screening the donor pigs and recipients include, along with the specific detection methods, either PCR-based, cell-based, or immunological methods, as well as the sample generation, sample preparation, sample origin, time of sampling, and the necessary negative and positive controls [62,70] (Table 7). The methods should be sensitive, specific, and should be validated as described [62].

**Figure 1:**
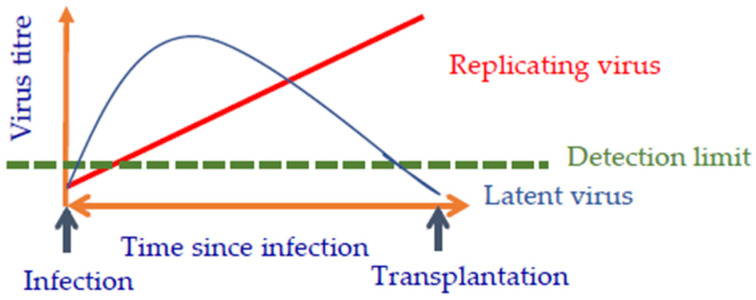
Differences in the detection of replicating and latent viruses.

It is important to know whether the virus is replicating or latent (Figure 1). Both replication-competent and latent viruses cannot be detected in the beginning of the infection, however they can be detected later. Whereas the amount of the replicating virus soon rises above the detection limit of the detection methodused, the latent virus goes into latency and cannot be detected any longer (Figure 1). However, after transplantation, the virus may be activated again, and replicates are consequently unrestricted in the transplanted pig organ and are able to harm the recipient. This exact scenario happened in the case of the first pig heart transplantation in Baltimore.

PCMV/PRV is an excellent example of a latent herpes virus. The infection normally happens early in life, and at that time, PCMV/PRV can be detected easily by PCR-based methods. In adult animals, when the virus is in its latent stage, it cannot be detected by PCR. However, the detection of antibodies as an indirect method to detect infection is possible [80]. It is important to note that young piglets may have antibodies against PCMV/PRV derived from the colostrum of their mothers, if they were positive [80]. Based on these results, a strategy should be selected to screen for PCMV/PRV in young and in old animals [80]. It is also important to note that PCMV/PRV is widely distributed, not only in production pigs, but also wild boars [79]. Therefore, pigs in facilities for xenotransplantation should not only be protected from contact with production pigs, but also with wild boars. Furthermore, for the generation of cloned and genetically modified pigs for xenotransplantation, oocytes and follicular fluid, which may be infected with PCMV/PRV, are used for somatic cell nuclear transfer (SCNT), and this may introduce the virus [115].

## 4. Elimination of Porcine Viruses

**Figure 2:**
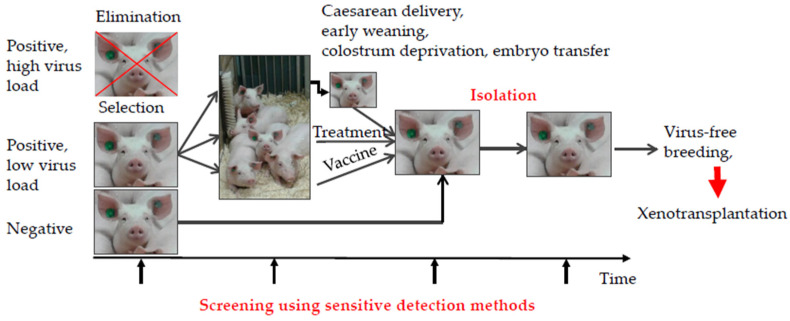
Elimination programs of potentially zoonotic pig viruses.

Most pig viruses can be eliminated from the pig herd (Figure 2). Negative animals can be used directly for xenotransplantation, whereas infected animals with a high virus load should be eliminated. In the case where no negative animals are available, animals with a low virus load should be selected, and the viruses can be eliminated by vaccination, if a vaccine is available, or by antiviral drugs, if available. If neither are available, viruses can be eliminated by early weaning to prevent transmission of the virus from the infected mother by milk. Viruses can be eliminated by colostrum deprivation or, in extreme cases, by caesarean delivery or embryo transfer (Figure 2). Once the virus is eliminated, the animal should be kept in isolation to avoid de novo infection or re-entry. Using sensitive detection methods, the animal should be screened before, during, and after elimination of the virus. Early weaning has been shown to be a successful approach for eliminating PCMV/PRV from pig herds [53,116].

Whereas most pig viruses can be eliminated using the above-mentioned strategies, PERVs, which are integrated in the genome of all pigs, cannot be eliminated this way. PERV-A and PERV-B are integrated in the genome of all pigs, PERV-C is present in many, but not all pigs. In addition, PERVs are active in their hosts, and their copy number increases with time (for review see [39]). This also leads to recombinations between PERV-A and PERV-C, the recombinant PERV-A/C viruses are characterized by high replication rates, and they are able to infect human cells. They are found integrated in the genome of certain somatic cells, but not in the germ line of the animals [35,117,118]. The isolation of replication-competent PERVs able to infect human cells (human-tropic) is rare [105]; It is important to note that PERV-A/C were found predominantly in minipigs [119].

Since PERVs are integrated in the pig genome, and cannot be eliminated as can all other viruses, several strategies have been developed in order to prevent the transmission of PERVs to the recipient (Table 8).

Since xenotransplantation surgeries are planned long in advance, the recipients could be vaccinated against PERV to prevent transmission, if necessary. At the moment, there is convincing evidence that no PERV has been transmitted in any transplantation or infection experiments in small animals, as well as in non-human primates, with or without pharmaceutical immunosuppression (for review see [12]). High titer antibodies neutralizing PERVs were produced, immunizing different species (goat, mice, rats, hamsters) with the recombinant ectodomain of the transmembrane envelope protein p15E and the entire surface envelope protein gp70 of PERV [120,121,122,123]. Since there is no animal model of a PERV infection available, in which the vaccine could be tested in vivo, we developed a model using the same principle vaccines based on the envelope proteins of the closely related feline leukaemia virus (FeLV), and showed that immunizing cats with our vaccine could protect the animals from a FeLV-induced leukaemia [136,137,138], suggesting that the PERV vaccine may also work in vivo.

**Figure 3:**
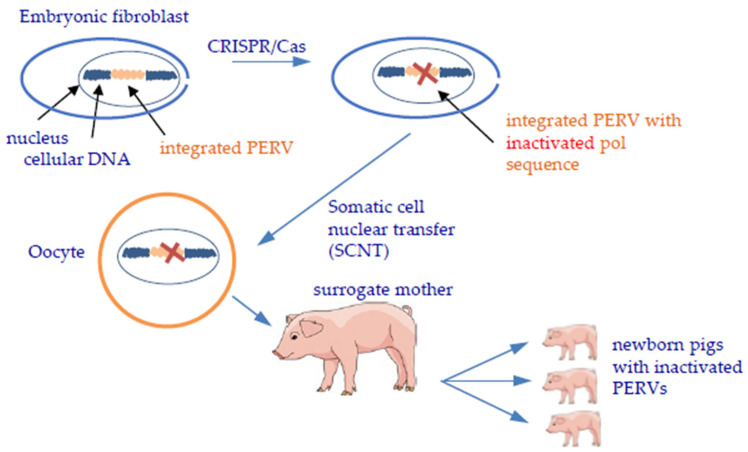
Inactivation of PERVs integrated in the pig genome using CRISPR/Cas and generation of piglets by somatic cell nuclear transfer (SCNT) [132].

Gene editing is a perfect way to inactivate PERVs in the genome. However, because the number of integrated proviruses may reach more than 60, there is risk that the numerous interventions in the cell genome may destroy the genome and kill the cell, as was observed when using the ZFN [133]. More successful was the inactivation of PERV using CRISPR/Cas [134,135] (Figure 3). Since PERV has not been transmitted until now, and since the off-target effects and the problems when breeding large numbers of CRISPR/Cas-treated animals are still unknown, it remains unclear whether this strategy should be used, or even if it can be used at all [41,139,140].

## 5. Future Clinical Trials

Based on the results of the first transplantation of a pig heart to a human patient, and on all preclinical trials performed with non-human primates, the following recommendations for future clinical trials are important [7]: First, in the study design, competent virologists should be involved. Second, the donor animals have to be analyzed with sensitive methods and appropriate strategies, especially in the case that latent viruses, such as herpes viruses, are under investigation. The Federal Drug Administration (FDA), with involvement from the Centers for Disease Control and Prevention (CDC) in the US, and the European Medicines Agency (EMEA) in Europe, must ensure that these methods are used.

## 6. Conclusions

Recent findings have shown that virus safety is as important for a successful xenotransplantation as the genetic modifications of the donor pig, the effectiveness of the immunosuppressive, and the skill of the surgeons. In the last several years, sensitive and specific methods to detect potential zoonotic pig viruses have been developed, and using these detection methods an elimination of most of theses viruses from genetically modified pig breeds generated for xenotransplantation, is possible. PERVs, which are integrated in the genome of all pigs, have not been transmitted in any of the many preclinical and clinical xenotransplantation trials performed so far, nor in any of the numerous experimental PERV infection experiments. To prevent PERV transmission after xenotransplantation, a range of different strategies has been developed, including the selection of PERV-C-free animals to prevent recombination between PERV-A and PERV-C. In order to prevent the transmission of latent viruses, mainly herpes viruses such as PCMV/PRV, appropriate sensitive detection methods and detection strategies must be used. In future clinical trials, competent virologists should be involved.

## Figures and Tables

**Table 1:** Reviews analyzing the potential impact of porcine viruses on xenotransplantation.

Viruses	Reviews
Different viruses, general aspects	Fishman [21], Yoo & Giulivi, 2000 [22], Takeuchi et al., 2005 [23], Mattiuzzo et al., 2008 [24], Scobie & Takeuchi, 2009 [25], Mueller et al., 2011 [26], Fishman et al., 2012 [27], Denner & Mueller, 2015 [28], Fishman, 2018 [29], Nellore & Fishman, 2018 [30], Fishman, 2020 [31],
Herpesviruses in general	Mueller & Fishman, 2004 [32], Tischer & Osterrieder, 2010 [33]
PCMV	Denner, 2015 [3], Denner, 2018 [2], Denner, 2022 [7]
PLHV	Denner, 2021 [10]
PERV	Wilson, 2008 [34], Denner, 2008 [35], Denner et al., 2009 [36], Denner & Tönjes, 2013 [37], Kimsa et al., 2014 [38], Denner, 2016 [39], McGregor et al., 2018 [40], Denner et al., 2018 [41], Denner, 2018 [12], Denner, 2021 [11],
Single stranded DNA viruses	Karuppannan & Opriessnig, 2018 [42]
Circoviruses	Denner & Mankertz, 2017 [43]
HEV	Denner, 2015 [8], Denner, 2019 [9]

**Table 2:** Viruses absent in Auckland Island pigs used for clinical islet cell xenotransplantations [47,48,49,50,51,52].

Virus Name	Abbreviation
Porcine circovirus 1	PCV1
Porcine circovirus 2	PCV2
Porcine lymphotrophic herpesvirus	PLHV
Porcine cytomegalovirus/porcine roseolovirus	PCMV/PRV
Rotavirus	RV
Porcine enterovirus type 1	PEV1
Porcine enterovirus type 3	PEV3
Porcine hemagglutinating encephalomyelitis virus	PHEV
Hepatitis E virus	HEV
Bovine viral diarrhea virus	BVDV
Suid herpesvirus 1 or Aujeszky’s disease virus or pseudorabies virus.	SuHV-1 or ADV or PrV
Porcine parvovirus	PPV
Porcine reproductive and respiratory syndrome virus	PRRSV
Porcine encephalomyocarditis virus	EMCV

**Table 3:** Microorganisms tested in the pig facility of the CiMM in Munich, Germany [53].

Testing	Microorganisms
Serological testing	*Actinobacillus pleuropneumoniae*, *Haemophilus parasuis*, *Lawsonia intracellularis*, *Leptospira* spp., *Mycoplasma hyopneumoniae*, *Pasteurella multocida*, porcine reproductive and respiratory syndrome virus (PRRSV), swine influenza virus (SIV), Porcine epidemic diarrhea (PED), Porcine respiratory coronavirus (PRCV), hepatitis E virus (HEV), transmissable gastroenteritis virus (TGEV)
Antigen testing	*Brachyspira hyodysenteriae*, salmonella, swine influenza virus
Fecal swabs	bacteriological content, endoparasites
PCR testing	*Lawsonia intracellularis*, *Brachyspira pilosicoli*, *Brachyspira hyodysenteriae*, hepatitis E virus (HEV), porcine cytomegalovirus/porcine roseolovirus (PCMV/PRV), rotavirus RV), coronavirus (CoV), tescho-sapelovirus
Cell culture	*Escherichia coli*, Salmonella group C
Parasites	Strongyloides

**Table 4:** Microorganisms excluded from a colony of designated pathogen free pigs [54].

Microorganisms	Species
Bacteria	*Actinobacillus pleuropneumonia*, *Actinobacillus suis*, *Bacillus anthracis*, *Bordetella bronchiseptica*, *Brucella* sp., *Campylobacter* sp., *Chlamydia* sp., *Erysipelothrix* sp., *Haemophilus parasuis*, *Lawsonia intracellularis*, *Leptospira* sp., *Mycoplasma hyopneumonia*, *Mycoplasma hyorhinis*, *Mycoplasma hyosynoviae*, *Mycobacterium tuberculosis*, *Mycobacterium bovis*, *Mycobacterium avium*, *Pasteurella multocida*, *Pasteurella. haemolytica*, *Salmonella* sp., *Brachyspira* sp., *Staphylococcus hyicus*, *Streptococcus suis*, *Yersinia* sp.
Fungi	Systemic mycoses including: *Blastomyces* sp., *Cryptococcus* sp., *Histoplasma* sp.
Parasites	Pathogeneic Protozoa including: *Cryptosporidium parvum*, *Giardia* sp., *Toxoplasma* sp., *Helminths*, *Trichinella spiralis*, Blood parasites
Arthropods	All pathogenic arthropods, e.g., lice and mite
Viruses	Porcine adenovirus, Bovine viral diarrhea virus, Porcine circoviruses 1 and 2, Encephalitis, Eastern and Western Equine, Encephalomyocarditis virus, Enterovirus, Hemagglutinating encephalomyelitis Virus, Hepatitis E virus, Infectious bovine rhinotracheitis Virus, Swine influenza virus, Porcine cytomegalovirus/porcine roseolovirus, Porcine parvovirus, Porcine reproductive and respiratory syndrome virus, Parainfluenza 3 Virus, Pseudorabies virus, Porcine respiratory coronavirus, Rotavirus, Transmissible gastroenteritis virus, Vesicular stomatitis virus (NJ & Indiana), West Nile fever virus, Porcine lymphotropic herpes virus 1 and 2

**Table 5:** Vaccines used in a pig breed generated for islet cell xenotransplantation [55].

Vaccine	Target Microorganisms	Manufacturer
ParaSail	*Haemophilus parasuis*	Newport Laboratories
CircoFLEX	Porcine circovirus 2 (PCV2)	Boehringer Ingelheim
MycoFLEX	*Mycoplasma hyopneumoniae*	Boehringer Ingelheim
Myco Shield	*Mycoplasma hyopneumoniae*	Novartis
Pneumostar SIV	H1N1 & H1N2 & H3N	Novartis
Enterisol Ileitis	*Lawsonia intracellularis*	Boehringer Ingelheim
Parvo Shield L5E	porcine parvovirus, *Erysipelothrix rhusiopathiae*, and *Leptospira canicola*, *grippotyphosa*, *hardjo*, *icterohaemorrhagiae*, and *pomona*.	Novartis
Prefarrow Shield 9d	Bordetella bronchiseptica, Clostridium perfringens type C, Erysipelothrix rhusiopathiae, K88, K99, 987P & F41 piliated E. coli, and Pasteurella multocida types A & D.	Novartis
Prosystem RCE	Two major Rotavirus serotypes, four major E. coli pilus antigens (K88, K99, F41 and 987P) and C. perfringens type C toxoid.	Merck
Ingelvac PRRS	PRRSV Stamm ATCC VR 2332 (Genotyp 2):	Boehringer Ingelheim
PRRS	PRRSV	Newport

**Table 6:** Publications describing methods for the detection of potential zoonotic porcine viruses in the context of xenotransplantation.

Viruses	Publication
General aspects	Chmielewicz et al., 2003 [63], Tucker et al., 2003 [64], Garkavenko et al., 2004 [50], Garkavenko et al., 2008 [49], Abrahante et al., 2011 [65], Wynyard et al., 2014 [51], Plotzki et al., 2016 [66,67], Gazda et al., 2016 [55], Morozov et al., 2016 [68,69], Denner, 2017 [70], Hartline et al. 2018 [71], Crossan et al., 2018 [72], Noordergraaf et al. 2018 [54], Krüger et al., 2019 [73], Matsumoto et al., 2020 [46], Denner 2020 [62], Halecker et al., 2021 [74]
PCMV	Mueller et al., 2002 [75], Mueller et al., 2004 [76], Morozov et al., 2016 [69], Plotzki et al., 2016 [77], Fiebig et al., 2018 [78], Hansen et al., 2022 [79], Halecker et al., 2022 [80]
PERV	Paradis et al., 1999 [81], Blusch et al., 2000 [82], Stephan et al., 2001 [83], Tacke et al., 2001 [84], Herring et al., 2001 [85], Denner 2003 [86], Nishitai et al., 2005 [87], Issa et al., 2008 [88], Xing et al., 2009 [89], Zhang et al., 2010 [90], Kaulitz et al., 2011 [91], Wynyard et al., 2011 [92], Xiang et al., 2013 [93], Kaulitz et al., 2013 [94], Semaan et al., 2013 [95], Guo et al., 2014 [96], Costa et al., 2014 [97], Gola & Mazurek, 2014 [98], Godehardt et al., 2015 [99], Morozov et al., 2017 [52], Mourad et al., 2017, [100], Li et al., 2017 [101], Fiebig et al., 2018 [102], Choi et al., 2017 [103], Kono et al. 2020 [104], Halecker et al., 2022 [105],
Circoviruses	Tucker et al., 2003 [63], Hattermann et al., 2004 [106], Karuppannan & Opriessnig, 2018 [42], Krüger et al., 2019 [107], Prinz et al., 2019 [108],
Single stranded DNA viruses	Karuppannan & Opriessnig, 2018 [42]
HEV	Busby et al., 2013 [109], Morozov et al., 2015 [110], Abicht et al., 2016 [111],
PLHV	Tucker et al., 2003 [63], Mueller et al., 2004 [75], Brema et al., 2008 [112], Issa et al., 2008 [88], Plotzki et al., 2016 [113]
Non-viral pathogens	Tönjes, 2018 [114]

**Table 7:** Components of the detection systems [62].

▪ Sensitive and specific detection methods
▪ PCR-based methods
▪ Cell-based methods
▪ Immunological methods.
▪ Sample generation
▪ Sample preparation
▪ Sample origin
▪ Time of sampling
▪ Negative and positive controls

**Table 8:** Strategies to prevent PERV transmission.

▪ Vaccine, based on neutralizing antibodies against the transmembrane and surface envelope proteins of PERV [120,121,122,123]
▪ Antiretroviral drugs [83,124,125,126,127,128]
▪ Reduction of PERV expression by siRNA [129,130,131,132]
▪ Gene editing
▪ Zinc finger nuclease (ZFN) [133]
▪ Clustered Regularly Interspaced Short Palindromic Repeats/CRISPR-associated 9 (CRISPR/Cas9) [134,135] (Figure 3)

## Data Availability

Not applicable.
